# Changing beliefs or changing behavior? Understanding the belief-to-behavior process and intervening to curb the impact of misinformation

**DOI:** 10.1002/jcpy.70014

**Published:** 2025-12-10

**Authors:** Hogeun Lee, Dolores Albarracín

**Affiliations:** University of Pennsylvania, Philadelphia, Pennsylvania, USA

**Keywords:** BDT, beliefs and lay theories, social influence and norms

## Abstract

A common assumption among scientists, journalists, and policymakers is that combating misinformation reliably changes behavior. However, the empirical evidence reveals that the belief–behavior association is often modest, variable, and context-dependent, raising critical questions about when and how to pursue belief versus behavior change. In this paper, we discuss the mechanisms by which beliefs influence behavior and the conditions under which addressing beliefs can change behavior. Specifically, we review the belief-to-behavior inference model, which proposes that beliefs influence behavior when (a) the belief is linked to a behavioral goal, (b) the inferential path from belief to behavior is relatively short, and (c) the belief–behavior association is preserved in memory. Our framework aligns intervention decisions with the cognitive architecture of belief–behavior correspondence and the intervention’s goals, whether maximizing belief accuracy or behavioral impact. We also reviewed individual and social-structural interventions that are best suited for changing beliefs versus behavior, conceptually integrating interdisciplinary work on behavior change with the psychology of belief change.

## INTRODUCTION

In an era shaped by pandemics, climate change, authoritarianism, and political polarization, evidence-based decision-making is critical. At the individual level, misinformation (i.e., false information) may lead to serious or irreversible consequences. At the social level, it can shape political and social decisions that undermine the public good and democratic functioning (Lewandowsky et al., 2012). However, what is the relation between belief and behavior? When should one try to change beliefs to change behavior, and when should one choose strategies that change behavior? What interventions should one use in each case?

The sense that we must fight misinformation to change behavior is common in science, journalism, and policy (Graves, 2016; Lazer et al., 2018). However, the empirical evidence for a strong, direct association between beliefs and behavior is underwhelming (Granados Samayoa & Albarracín, 2025b). A recent synthesis of meta-analyses demonstrated that the average correlation between belief and behavior often falls below the conventional threshold for a medium-sized effect (Albarracín, Fayaz-Farkhad, & Granados Samayoa, 2024). In short, while beliefs may contribute to behavior, the average belief–behavior correlation is *r* = 0.17, corresponding to belief accounting for 3% of the behavior variance (Albarracín, Fayaz-Farkhad, & Granados Samayoa, 2024). The variability of these correlations is also considerable (range in *r* = 0.03–0.22; Albarracín, Fayaz-Farkhad, & Granados Samayoa, 2024).

Given the size and variability of the belief-behavior correlation, the decision to combat misinformation should depend on whether the goal is to mitigate effects on beliefs (e.g., the belief that vaccines contain mercury) versus effects on behavior (e.g., vaccination). Educators and journalists must ensure accurate beliefs, even if the beliefs have no behavioral consequence. Educators must convey accurate information in various domains, from math to language to science. Journalists must promote factual accuracy, regardless of the downstream behavioral effects of news (Graves & Amazeen, 2019; Kovach & Rosenstiel, 2007). In contrast, public health officials must increase health screenings and preventive behaviors, in which case, curbing misconceptions should be a goal only if those beliefs drive behavior.

This review begins with a model of the psychological mechanisms connecting belief to behavior. We first describe how people make a belief-to-behavior inference (Granados Samayoa & Albarracín, 2025b) and use our model to predict when interventions should try to change beliefs to change behavior instead of simply changing beliefs. We then examine a range of intervention strategies, including individual- and societal-level interventions foreign to the psychology of belief (Albarracín, Fayaz-Farkhad, & Granados Samayoa, 2024). This paper addresses two questions: (1) When should we target belief versus behavior? and (2) what interventions should we use to change belief versus behavior. The questions are both conceptual and practical, and have implications for psychology, marketing, communication, and other fields (see also Albarracín & Zhou, 2025).

### The relation between beliefs and behavior and when to target beliefs versus behavior

Understanding belief and behavior is essential for designing effective communications and interventions to combat misinformation. Beliefs are defined as probability judgments that link a referent, such as a behavior, object, or concept, to an attribute, outcome, or property (Fishbein & Ajzen, 1975; Granados Samayoa & Albarracín, 2025b; Wyer & Albarracín, 2005). This probabilistic function distinguishes beliefs from related constructs such as attitudes, which involve evaluations, and values, which are evaluations of abstract entities such as freedom (Albarracín, Fayaz-Farkhad, & Granados Samayoa, 2024; Granados Samayoa & Albarracín, 2025b; Wyer & Albarracín, 2005). Whereas beliefs often operate as upstream cognitive inputs, behavior is often the downstream output of the belief–attitude–intention sequence (Albarracín, Fayaz-Farkhad, & Granados Samayoa, 2024; Fishbein & Ajzen, 1975, 2010). Behavior is broadly defined as an observable action or inaction performed by an individual in a specific context (Albarracín, Fayaz-Farkhad, & Granados Samayoa, 2024; Granados Samayoa & Albarracín, 2025b).

As mentioned, whether the goal is to shape beliefs or behavior is critically important to the problem of addressing misinformation. Somebody focused on accuracy, such as a fact-checker, must ensure accurate beliefs. However, somebody interested in changing behavior must decide whether to target beliefs or behavior. Fortunately, a recent theoretical model about belief-to-behavior inferences (Granados Samayoa & Albarracín, 2025b) can inform this decision.

According to Granados Samayoa and Albarracín (2025b), individuals form beliefs either in isolation or as part of a probabilistic inferential chain that connects those beliefs to behavioral attitudes and intentions. Forming a belief does not translate into behavior unless accompanied by reasoning about its practical consequences. For example, consider A as believing that “a vaccine causes severe side effects,” and C as the behavioral decision “I will not take the vaccine.” A key insight from the model is that if no belief-to-behavior inference has been formed, then A does not serve as the cognitive basis for C. Consequently, attempts to change C by targeting A are likely to fail even if the behavior C seems like a natural consequence of A. Instead, we must direct attention to the behavior to change it.

Although prior theories have emphasized the role of beliefs as precursors of behavior, they have generally conceptualized beliefs as distal causes of behavior, assuming that beliefs are aggregated into expectancy-value summaries or similar cognitive algebra (Anderson, 1974; Fishbein & Ajzen, 1975, 2010). Thus, these past models have proposed that beliefs aggregate, without fully considering whether, when, or how specific beliefs are activated and influence behavior (Granados Samayoa & Albarracín, 2025b). Also, prior models (e.g., Fazio & Olson, 2014) have not articulated that people need a conscious behavioral goal, that belief-to-behavior inferences are not formed automatically. Although automaticity controls many facets of mental life (e.g., Bargh et al., 2001), including later activation of goals, we posit that the initial goal formation is conscious. Moreover, given the probabilistic nature of the belief-to-behavior inference (Jacobs & Kruschke, 2010), different beliefs could produce diverse, even contradictory behaviors. By contrast, the idea of aggregation assumes that the contradictions are resolved.

**FIGURE 1:**
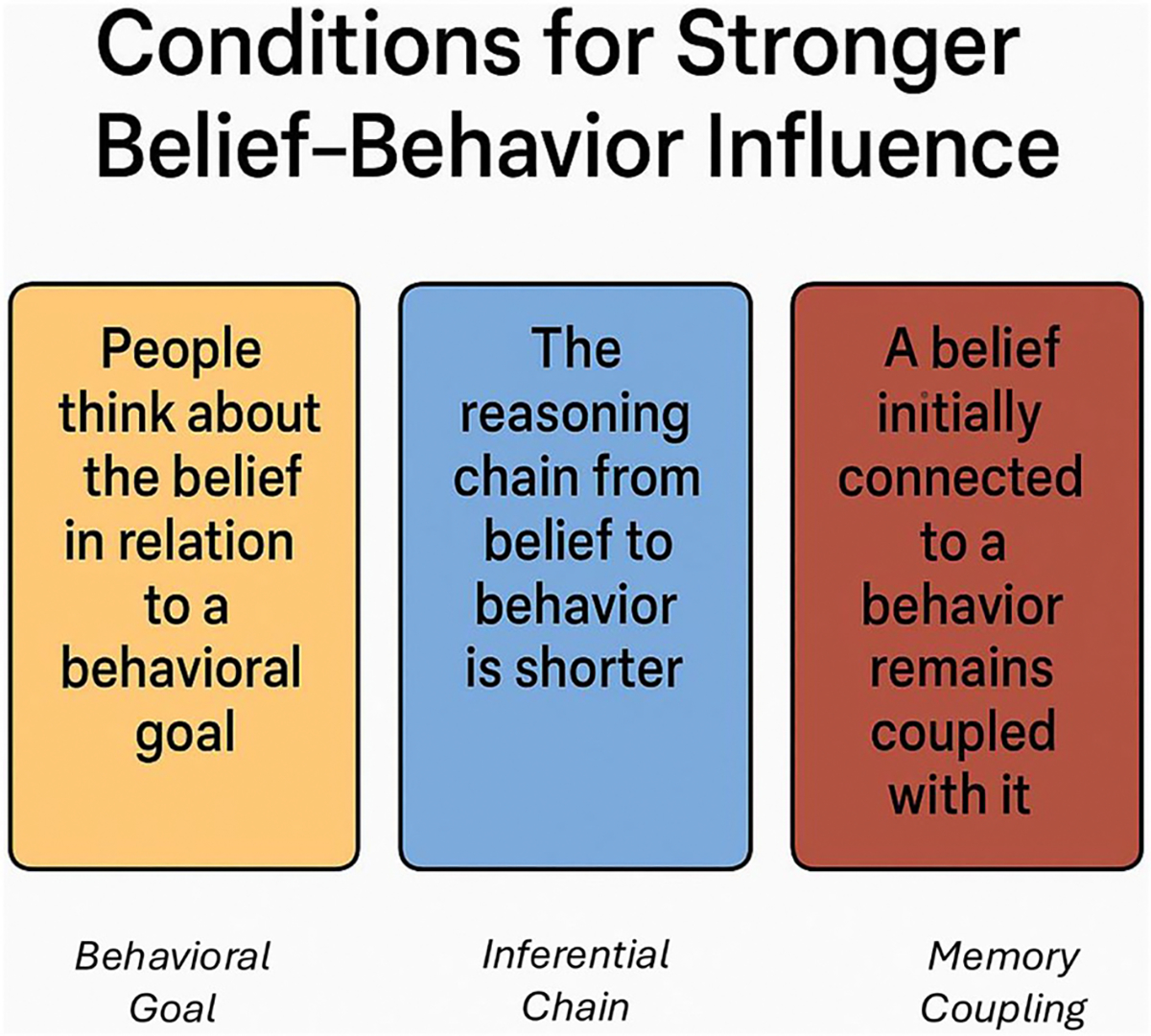
Conditions that promote belief-to-behavior influence according to the belief-to-behavior inference model.

Critical to this analysis, this model also specifies how belief-to-behavior inferences operate, thus illuminating when a belief influences behavior. Specifically, in this paper, we infer three implications that can help to decide when to target belief as a way of changing behavior. These three conditions appear in Figure 1 and are as follows.

1. A belief is likely to influence behavior when people think about it in relation to a behavioral goal.
2. A belief is more likely to influence behavior when the reasoning chain from belief to behavior is relatively short.
3. A belief is likely to influence behavior when the belief was initially connected to a behavior, and the connection is maintained in memory.

The conditions in Figure 1 offer both common sense and novelty. On the one hand, the requirement of a behavioral goal is obvious. On the other hand, prior models that introduced goals (Ajzen & Kruglanski, 2019) have focused only on the goal of procurement, leading to attitudes, and the goal of approval, leading to social norms. Thus, the fundamental process that initiates a connection between belief and behavior remains undertheorized.

The following sections elaborate on the conditions presented in Figure 1. Following that, we present possible intervention strategies that focus on beliefs or behavior. In doing so, we take a fresh look at the problem of misinformation and bring together a dispersed literature, making a novel contribution to the misinformation and belief change areas.

### Presence of a behavioral goal

The belief-to-behavior inference model identifies motivational and cognitive factors that determine these inferential processes (Granados Samayoa & Albarracín, 2025b). It proposes that belief-to-behavior inferences are formed when individuals have activated behavioral goals—that is, when they are oriented toward making a decision or executing an action, rather than merely acquiring information. Thus, a close belief-to-behavior correspondence requires a behavioral goal, not just any motivation, and cannot occur unless individuals deliberate on the behavioral goal. One indirect piece of supportive evidence comes from research on accuracy prompts on social media. Although accuracy prompts effectively enhance people’s ability to distinguish accurate from false information, they have minimal impact on the behavior of sharing false content (Arechar et al., 2023). This finding thus suggests that elaborating on one’s belief is insufficient to change behavior (Granados Samayoa & Albarracín, 2025b).

### Shorter belief to behavior distance

The belief-to-behavior inference model also posits that the strength of the connection between a belief and a behavior is influenced by the length of the inferential chain that links belief to behavior (Granados Samayoa & Albarracín, 2025b). Specifically, shorter inferential paths are more likely to yield consistent behavioral outcomes. Outcome beliefs involve probability judgments about specific consequences and require only two inferential steps. For example, the belief that “a vaccine causes severe side effects” may prompt the evaluative judgment, “if a vaccine causes severe side effects, it is harmful.” From there to the behavioral intention, “if it is harmful, I will not take it.” By contrast, existence beliefs, which concern whether something exists, and descriptive beliefs, which concern the attributes of an entity, can generate a behavioral conclusion only after more extensive reasoning chains (Granados Samayoa & Albarracín, 2025b). Consider the belief that “large pharmaceutical companies fabricated the pandemic.” On its own, this belief does not immediately prescribe any specific behavior. To reach a behavioral conclusion, one might first infer, “if the pandemic was fabricated, it is not a real danger,” which may lead to “if it is not a real danger, the vaccine is unnecessary.” Finally, “if the vaccine is unnecessary, I will not take it.” Each additional inferential step introduces cognitive demands and uncertainty, weakening the overall belief-behavior link.

Empirical evidence lends support to this notion. A reanalysis of data from Albarracín, Fayaz-Farkhad, and Granados Samayoa (2024), conducted by Granados Samayoa and Albarracín (2025b), revealed that outcome beliefs demonstrated significantly stronger correlations with behavior (average *r* = 0.30; *r* = 0.26 after ruling out extreme publication bias), and interventions to change outcome beliefs produced a greater behavioral impact (*r* = 0.19). In contrast, existence beliefs, such as those involving conspiracy theories or religious propositions, exhibited tiny associations with behavior (average *r* = 0.12; *r* = 0.09). Descriptive beliefs, including those related to stereotypes or mindset theories, also showed minimal correlations (average *r* = 0.12; *r* = 0.13 after ruling out extreme publication bias), and interventions targeting these beliefs yielded even more minor behavioral effects.

### Retaining the belief-behavior coupling in memory

The belief-to-behavior model also states that behavioral attitudes, intentions, and habits can separate from the beliefs that initially produced them (Granados Samayoa & Albarracín, 2025b). As a result, changing beliefs is not always sufficient to change behavior. Even when misinformation is successfully corrected at the belief level, its influence on attitudes and behavioral intentions can persist (Johnson & Seifert, 1994; Swire et al., 2017; Thorson, 2016).

The principle of memory coupling yields two critical implications for addressing misinformation. First, ideally, belief-targeted interventions must be implemented early, before the misinformation gives rise to more stable constructs such as attitudes, intentions, or proceduralized behavioral inferences. Second, once attitudes and intentions govern behavior decoupled from belief in memory, belief correction alone will be moot. Effective interventions may still be possible at that stage, but will require interventions that address attitudes, social norms, habits, and other immediate behavioral precursors. This is what behavioral interventions are about, a topic we discuss in the coming sections.

## INTERVENTIONS TO CHANGE BELIEF VERSUS BEHAVIOR

In the previous section, we discussed when beliefs will promote behavior versus fail to do so. Based on that discussion, targeting belief is appropriate when belief change is the goal and the conditions in Figure 1 are met. In contrast, targeting behavior more directly is indicated when one wants to change behavior, but the conditions in Figure 1 are unmet. But how should one approach belief and behavioral change? To answer this question, in the next sections, we discuss different interventions investigated in two kinds of literature: (a) the literature about belief change and (b) the literature about behavior change.

**FIGURE 2:**
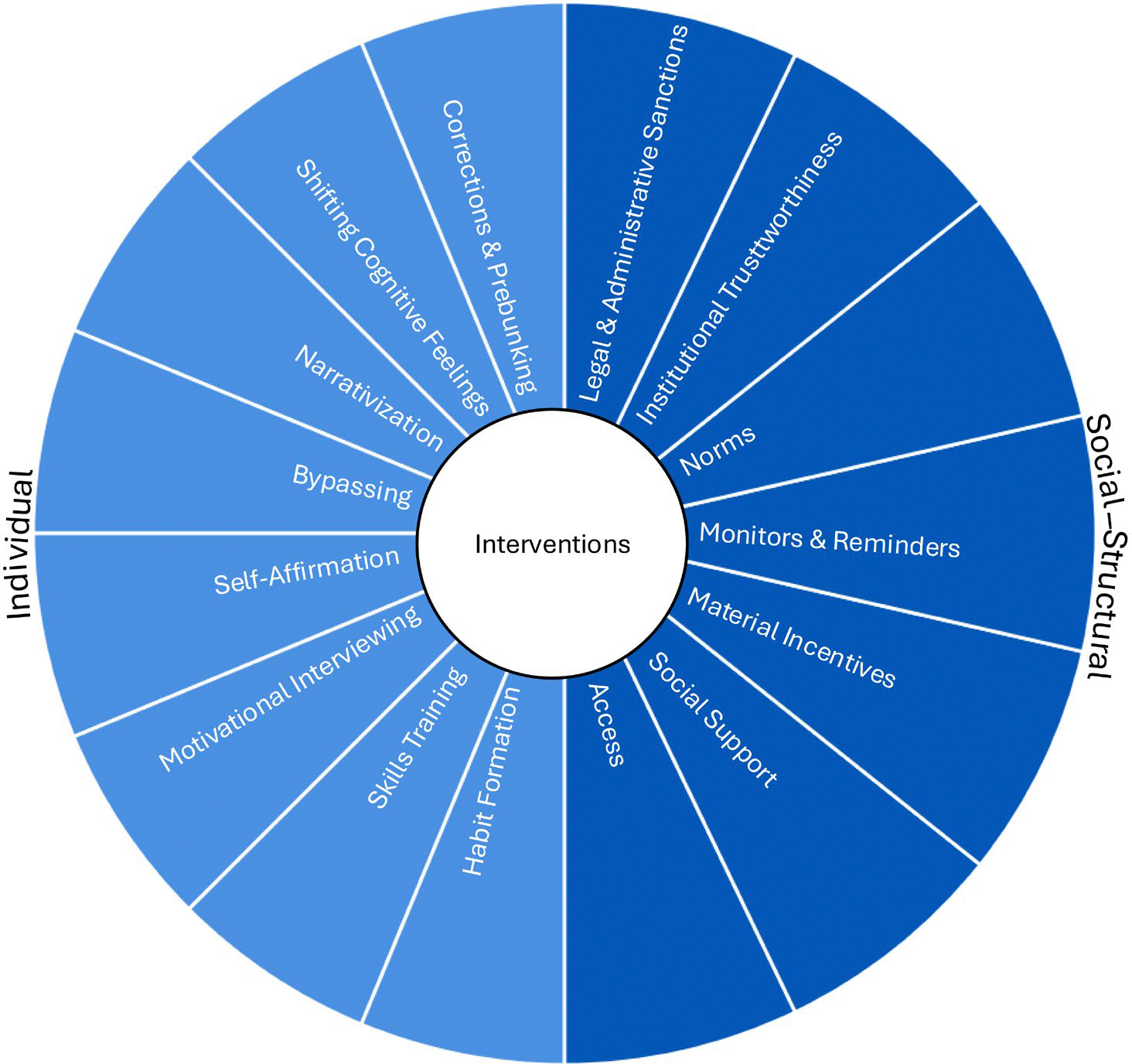
Interventions to change beliefs and behavior relevant to misinformation.

In our discussion about interventions to change beliefs versus behavior, we follow Albarracín, Fayaz-Farkhad, and Granados Samayoa’s (2024) review, which classified intervention targets into individual and social-structural. However, the classification pertained exclusively to interventions examined in the context of behavioral change. As such, that synthesis did not consider misinformation or belief change as the intervention goal. Meanwhile, the psychology of belief and misinformation has focused exclusively on belief-change interventions. For example, many prior studies have assessed corrections and accuracy prompts, rather than conceptualizing how to change behavior. Therefore, in this paper, we extend Albarracín, Fayaz-Farkhad, and Granados Samayoa’s (2024) framework to include belief-change interventions. The proposed interventions, which appear in Figure 2, also include individual and social-structural strategies.

Individual factors fall under the actor’s control, without requiring other people. Someone can acquire knowledge about an object without much intervention from others. Individual intervention targets include knowledge, attitudes, general skills, beliefs, emotions, behavioral skills, and habits (Albarracín, Fayaz-Farkhad, & Granados Samayoa, 2024). In the context of misinformation, individual interventions that address knowledge and beliefs involve corrections, defined as factual refutations of a misconception (Chan & Albarracín, 2023; see also prebunking, van der Linden & Roozenbeek, 2020); approaches that capitalize on cognitive feelings, defined as subjective experiences of familiarity and fluency (Jacoby & Dallas, 1981; Schwarz, 2015; Zajonc, 1968); as well as narrativization (Green & Brock, 2000). Interventions that address attitudes and emotions include self-affirmation (Steele, 1988), motivational interviewing (Rollnick & Miller, 1995), behavioral skills training (Bandura, 1997) involving training in defensive skills around misinformation (Roozenbeek, van der Linden, et al., 2022); and interventions to create habits (Wood & Neal, 2007).

Social and structural factors involve other people, groups, institutional actors, group and institutional symbols, or social influence (for the impact of the environment, Albarracín & Dai, 2024). Social-structural interventions involve modifying legal and administrative regulations (Asch & Rosin, 2016), increasing the trustworthiness of a system (Tyler, 2006), changing social norms (Rhodes et al., 2020; Yamin et al., 2019), introducing monitors and reminders (Cohen & Andrade, 2018; Latkin et al., 2010), supplying material incentives (Ariely et al., 2009; Galbiati & Vertova, 2014), and providing social support for a behavior (Carron et al., 1996; DiMatteo, 2004). Social-structural interventions also involve access and defaults (Thaler & Sunstein, 2008), which have been extensively researched not only in behavioral economics but also in psychology and other fields (Brewer et al., 2017; Everett et al., 2015; Lehmann et al., 2016; White et al., 2021).

**FIGURE 3:**
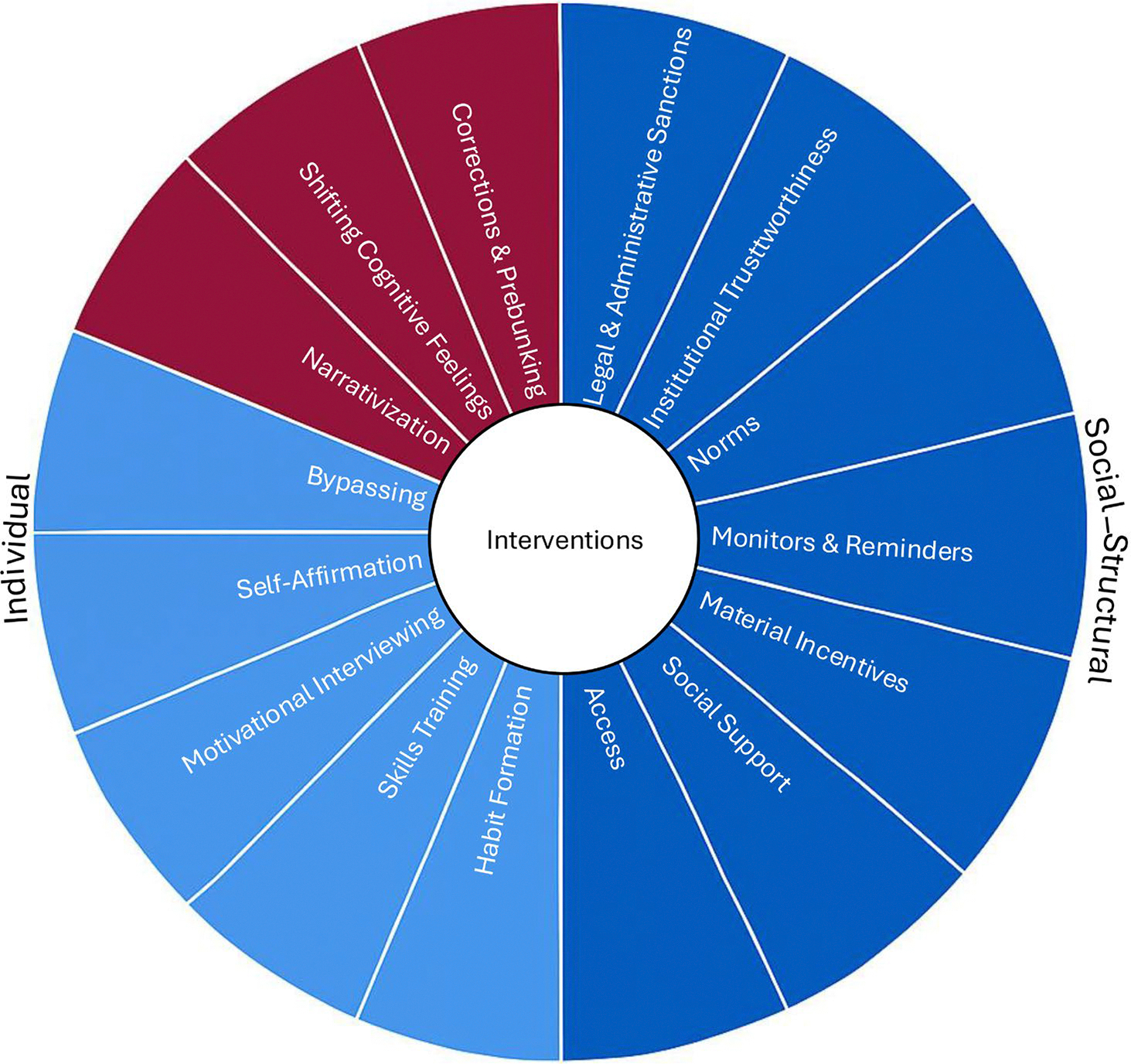
Interventions appropriate for belief change (in red) versus belief and behavioral change (in blue).

In the following sections, each intervention is discussed in terms of its theoretical foundation and role in influencing beliefs versus behavior. Figure 3 separates interventions most appropriate for belief change (in red) versus those most appropriate for behavioral change (in blue). Thus, even though belief and behavior change may be possible with each intervention, interventions that target belief directly are deemed most appropriate for belief change, whereas those that target behavior are deemed most appropriate for behavior change. In practice, behavior and its antecedents, such as attitudes, can have effects on beliefs, although such indirect effects are more likely to be weaker (Albarracín, 2002).

### Individual-level interventions

As mentioned, individual interventions include correction and prebunking, shifting cognitive feelings, narrativization, bypassing, self-affirmation, motivational interviewing, skills training, and programs to promote habits (see Figure 2). Below, we discuss existing research about each strategy, with particular attention to belief and behavior change.

### Correction and prebunking

One of the most consistently supported strategies to fight inaccurate beliefs is providing rebuttals and detailed refutations of the misinformation (Chan & Albarracín, 2023; Chan et al., 2017; Walter & Murphy, 2018; Walter & Tukachinsky, 2020; but see Walter et al., 2021 for an exception). This approach stems from mental model theory (Johnson-Laird, 1994, 2005), which posits that people construct coherent representations of events to make causal sense of information. When corrections fail to offer an alternative model, individuals are often unwilling to discard the original misinformation, relying on an incorrect but complete model over an incomplete one (Ecker, Lewandowsky, et al., 2022; Johnson & Seifert, 1994). Effective corrections, therefore, should not only refute false claims but also supply coherent, explanatory content that fills the gaps left by the retracted information.

A crucial implication of the mental model notion is that the effectiveness of corrections is influenced by how deeply individuals process the misinformation (see also Petty & Cacioppo, 1986). When recipients elaborate on the false claim by generating causal explanations or mentally simulating outcomes, they may form entrenched mental models that make later corrections harder to accept (Chan et al., 2017). For example, after being presented with fictitious case studies suggesting that risk preference predicts success as a firefighter, people continue to believe this theory even after being told that the case studies were fabricated. This continued influence is particularly strong among those who were explicitly instructed to explain the relations they identified (Anderson et al., 1980). The more compelling and elaborate the misinformation, the more coherent and detailed the correction must be to succeed. Addressing the entirety of the inaccurate narrative is important for other reasons as well. The implied truth effect suggests that when only parts of a statement are explicitly corrected, individuals may infer that the uncorrected portions are accurate (Pennycook et al., 2020).

Although correction strategies are successful at changing beliefs, their impact is often limited by the continued influence of misinformation (Davies, 1997; Jelalian & Miller, 1984). Given these challenges, a better way to mitigate the impact of misinformation is not a remedy after the exposure, but prevention. This preventive approach is best illustrated by *prebunking* (van der Linden & Roozenbeek, 2020), a strategy grounded in inoculation theory (McGuire, 1961, 1964; Traberg et al., 2022). Analogous to biological immunization, exposing individuals to a weakened version of persuasive misinformation, accompanied by an opportunity to counterargue, can prevent belief in misinformation later.

Even though correction is most appropriate for belief change, corrections may have uses when the goal of an intervention is to influence behavior. When a belief is well-connected to a behavior, as is the case for an outcome belief, introducing a detailed correction is likely beneficial. However, corrections might also induce a behavioral goal by highlighting the behavioral relevance of a belief correction (see Figure 1). For example, in a study conducted in the context of a health crisis, detailed messages containing a list of preventive behaviors heightened not only perceived severity (i.e., beliefs) but also behavioral intentions (van der Meer & Jin, 2020). This finding supports the idea that corrections can influence behavioral intentions, and possibly behavior, when they include concrete, actionable recommendations that ensure the desired belief-to-behavior inference (see Albarracín et al., 2018). They can also achieve this objective when they discuss outcome evaluations (see e.g., Fishbein et al., 1999).

A more subtle yet valuable approach to changing behavior via belief change may be to leverage the principle of compatibility. According to it, when two measures (e.g., an attitude and a behavior) are similar in target (e.g., bread), action (e.g., buying), context (e.g., the grocery store), and time (e.g., today), the stronger their correlation should be (Fishbein & Ajzen, 2010). For instance, environmental context affects decision-making, as when polling in schools increases support for school funding initiatives (Berger et al., 2008). Hence, delivering corrections at moments and in locations that enhance behavioral relevance (Glasman & Albarracín, 2006), such as correcting misinformation about mask-wearing at the entrance of a public building may be more effective than doing the same on television in the middle of the night.

### Shifting cognitive feelings

A meta-cognitive approach to misinformation correction focuses on how subjective experiences of information fluency (i.e., ease of processing a particular content) and familiarity (i.e., the sense that we have encountered the content before) can influence beliefs (Jacoby & Dallas, 1981; Schwarz, 2015; Zajonc, 1968). When a message is easy to process due to repetition, simplicity, or coherence, it often feels more credible, regardless of its factual accuracy (Reber & Schwarz, 1999; Schwarz, 2015). This phenomenon, known as the *illusory truth effect*, explains why both accurate and inaccurate statements can become more persuasive with repetition, especially when recipients are distracted or have limited cognitive resources (Sanderson et al., 2023).

Even though repeating corrections can give rise to feelings that reinforce the correction, efforts to correct misinformation can sometimes backfire by reinforcing the misinformation’s familiarity (Pluviano et al., 2017; Skurnik et al., 2005; for a review, see Albarracín, Oyserman, & Schwarz, 2024). According to the notions of schemas and tags (Clark & Chase, 1972; Schmidt & Sherman, 1984), negated information is encoded by linking an affirmative proposition to a false tag (e.g., “Vaccine is dangerous” + “not”). Over time, the tag can be lost, especially under cognitive strain (Kumkale & Albarracín, 2004), leaving behind only the affirmative proposition along with feelings of familiarity. Although the backfire effect is not common (Walter & Tukachinsky, 2020), the risk remains, particularly when misinformation is repeated without clear disconfirmation or warning cues (Ecker, Lewandowsky, et al., 2022; Lewandowsky et al., 2012).

From the perspective of the belief-to-behavior inference model, many beliefs shaped by meta-cognitive factors lack immediate behavioral relevance and may persist in memory as passive, unelaborated constructs. However, beliefs that are disjointed from a discounting cue may later be retrieved in contexts that elicit behavioral goals. For instance, a familiar but false belief about vaccine safety may have no effect until someone confronts the decision to vaccinate, when the belief can become part of a behavioral inference.

### Narrativization

Another strategy for changing beliefs is narrativization. Unlike purely statistical or factual messages, narratives organize information into a coherent, emotionally engaging structure that can resonate with audiences on both cognitive and affective levels (Green & Brock, 2000; Kopfman et al., 1998). By telling stories that include characters, goals, actions, and outcomes, narratives invite readers to experience events in an emotional way. This immersive quality, known as *narrative transportation* and identification with characters, makes recipients less able and motivated to generate counterarguments (Green & Brock, 2000; Moyer-Gusé, 2008; Ratcliff & Sun, 2020). Therefore, narratives could reduce resistance in contexts that may otherwise prompt resistance.

Despite the strong theoretical rationale for the use of narrative correction, the empirical evidence regarding its effectiveness remains inconclusive. Experiments reporting null findings rely on short-form fact-checking messages typical of social media, contexts that may limit transportation or identification with characters. For instance, the narrative stimuli used by Ecker et al. (2020), who found no effects of narratives, primarily consisted of interviews and testimonials, which function more as exemplars (Zillmann, 1999) than narratives (see Bigsby et al., 2019 for this distinction). In contrast, studies reporting positive effects of narratives often compare basic narrative formats with messages with other persuasive elements, using a no-correction control instead of a non-narrative correction control (Sangalang et al., 2019; Walter et al., 2019). Therefore, even if narratives outperform non-narrative messages in some cases, the findings have limited practical relevance.

Even if narratives are not advantageous for belief change, they may still have value for behavior change. Beyond merely reducing resistance, narratives enable vicarious experiences, allowing individuals to mentally simulate scenarios as if they lived them and behaviors if they had enacted them or wanted to do so. As a result, narrative corrections may not only change beliefs but also promote mental simulations of behavior that, in turn, influence beliefs via self-perception (Albarracín & Wyer, 2001; Bem, 1965, 1972). This process aligns with the belief-to-behavior inference model, which also considers the reverse inference where previous behaviors can shape later beliefs and attitudes (Granados Samayoa & Albarracín, 2025b). In fact, a meta-analysis supported this point indirectly, showing that non-narrative (i.e., statistical) evidence affected beliefs and attitudes, whereas narrative evidence affected behavioral intentions (Zebregs et al., 2015). Given these considerations, the behavioral impact of narratives should be further examined with actual behavioral measures.

### Bypassing

Bypassing is an indirect approach that, rather than directly confronting misinformation, highlights beliefs with opposite evaluative implications about the focal object to indirectly shift attitudes and behavioral intentions with the goal of changing behavior (Calabrese & Albarracín, 2023; Granados Samayoa & Albarracín, 2024, 2025a). For example, instead of refuting the claim that genetically modified (GM) foods are harmful (e.g., “GM foods cause severe allergic reactions”), a bypassing strategy would highlight the benefits of GM foods (e.g., “GM foods alleviate world hunger”). In such studies, participants exposed to bypassing messages form more positive attitudes toward GM foods and are less willing to support restrictive GM food policies than those exposed to misinformation alone. Notably, bypassing is as effective as direct corrections in reducing the influence of misinformation on attitudes and intentions, with a key difference: corrections reduce belief in misinformation, whereas bypassing changes attitudes without directly altering the inaccurate belief (Calabrese & Albarracín, 2023). Subsequent studies using within-subjects designs have found that bypassing is even more effective than correction in shifting policy attitudes and behavioral intentions in the context of short messages (i.e., news headlines; Granados Samayoa & Albarracín, 2025a).

Although bypassing research is still developing and should be assessed with behavioral change, initial findings provide valuable insights. When message recipients have belief formation goals, bypassing outperforms correction. However, when individuals have attitude formation goals, bypassing does not surpass correction and both methods are equally effective (Granados Samayoa & Albarracín, 2025a). Thus, bypassing strategies may be especially advantageous when attitudes are not yet formed or when prior attitudes are not activated and salient. Once attitudes have been formed, any belief about the issue can recall these attitudes, promoting attitude persistence.

A consideration for future research is that past studies on bypassing have primarily involved inaccurate outcome beliefs. If the misinformation involves existence or descriptive beliefs rather than outcome beliefs, bypassing might prove even more effective than correction because the behavioral inference will be formed anew.

### Self-affirmation

Many attitudes and behaviors persist because they support important notions about the self or the world, rather than for objective reasons (Kunda, 1990; Taber et al., 2009; Walter & Tukachinsky, 2020). As a result, one promising strategy to change behavior is self-affirmation. Self-affirmation theory posits that recalling and affirming a person’s core values can reduce the need to process information to defend the self (Correll et al., 2004; Sherman et al., 2020; Steele, 1988). For example, when it comes to behavioral change, self-affirmation has small positive effects on health behaviors (Epton et al., 2015; Sweeney & Moyer, 2015). One caveat is that self-affirmation may need to become behaviorally relevant, a process that may take time. For example, vicarious self-affirmation does not influence the intention to quit smoking e-cigarettes right after a correction, but it has delayed effects on intentions (Walter et al., 2019). Presumably, people change their beliefs through self-affirmation, but behavioral intentions change later, when, for example, a smoker reflects on whether to continue to smoke.

Although not its initial focus, self-affirmation has been used to support belief change as well. For example, individuals who complete a self-affirming task are more likely to accept corrections and abandon false beliefs, even when those beliefs are identity-relevant (Carnahan et al., 2018). People also change their beliefs about e-cigarettes when their beliefs are challenged after they observe a character who experiences personal success (Walter et al., 2019). Thus, even though self-affirmation is traditionally associated with behavior change, it may be used to change beliefs as well.

### Motivational interviewing

Motivational interviewing is a client-centered communication approach designed to enhance individuals’ intrinsic motivation and commitment to behavior change. The method promotes self-guided reflection that acknowledges the ambivalence people feel about changing a behavior (Rollnick & Miller, 1995; Rubak et al., 2005). It emerged as an alternative to directive recommendations, which often provoke defensiveness, and has been inspired by cognitive dissonance (Festinger, 1957; Brehm, 1966; for the theoretical basis of motivational interviewing, see Dowd & Sanders, 1994; Whitehead & Russel, 2004). Motivational interviewing builds collaboration between a counselor and a participant and affirms the participant’s autonomy, allowing individuals to explore their own reasons for change in a supportive, nonjudgmental context. The method emphasizes not only the reasons to change but also the motivations that prevent the change, without overtly pushing participants to change.

A robust body of evidence has demonstrated that motivational interviewing promotes health-related behavior change (Burke et al., 2004; Gayes & Steele, 2014; Jensen et al., 2011). For example, motivational interviewing was implemented with teenagers suffering from diabetes, whereas the control group received supportive medical visits (Channon et al., 2007). This randomized controlled trial showed that serum A1C showed greater improvements in the group receiving motivational interviewing compared with the control condition.

Although originally developed for clinical and therapeutic settings, the method can curb behaviors relevant to misinformation as well as misconceptions. For instance, in contexts like vaccine hesitancy, where misinformation is widespread, motivational interviewing allows practitioners to correct misconceptions. A typical strategy involves first asking individuals what they already understand, then respectfully offering new information, and finally eliciting their thoughts on the updated information (Boness et al., 2022).

### Skills training

Behavioral skills, defined as routines that enable individuals to perform specific target behaviors, have been shown to correlate with actual behavior (Albarracín, Fayaz-Farkhad, & Granados Samayoa, 2024; Bandura, 1997). In the health context, interventions such as teaching communication skills, providing arguments to resist unsafe sex or smoking, and engaging in role-play exercises have proven effective in driving behavioral change (Albarracín et al., 2003; Duncan et al., 2000; Fisher et al., 2002).

Although less frequent, skills training has been used to change beliefs. A relatively new strain of inoculation strategy involves highlighting the deceptive rhetorical tactics or logical fallacies that commonly accompany misinformation, rather than directly counterarguing specific misinformation (Banas & Miller, 2013; Lewandowsky & van der Linden, 2021; van der Linden & Roozenbeek, 2020). For example, short prebunking videos that warn viewers about manipulation techniques not only improve their ability to distinguish accurate information from misinformation but also improve their perceived behavioral control and the quality of their sharing decisions (Roozenbeek, van der Linden, et al., 2022). Also, a game developed by Roozenbeek and van der Linden (2019) involves role-playing as a fake news creator whose goal is to gain as many followers as possible without losing credibility. Players thus become familiar with the deceptive rhetorical strategies and logical fallacies commonly found in misinformation. They can then discern information accuracy and form intentions to share accurate information more than inaccurate information (Roozenbeek, Traberg, & van der Linden, 2022; Roozenbeek & van der Linden, 2019).

### Habit formation

According to the notion of habit, repeating any routine can automate it to the point that the behavior can continue in the absence of rewards (Ouellette & Wood, 1998). Most of the work on habits concerns overt behavior. For example, past recycling and past vaccination predict future recycling and vaccination (Albarracín, Fayaz-Farkhad, & Granados Samayoa, 2024). Based on these findings, behavioral change interventions have tried to automate behavior to achieve sustainable behavior changes. For *dietary behavior, stop signal training, attentional bias modification, and implementation intentions can change behavior. H*abit reversal training for tic reduction has been very successful as well (Wile & Pringsheim, 2013). For instance, if stress or hunger increases tics, the tic can be eliminated by repeating movements that are incompatible with the tic (Wile & Pringsheim, 2013).

Sharing information on social media illustrates how sharing behavior automates as well (Ceylan et al., 2023). In Ceylan et al. (2023), social media users reported regularly sharing messages they perceived to be false or inconsistent with their beliefs. Furthermore, the relation between accuracy cues and information sharing can be automated through interventions. When that occurs, habitual sharers asked to think about accuracy are more likely to share accurate information than nonhabitual ones (Ceylan et al., 2023).

### Social-structural interventions

The prior sections described individual interventions that are best poised to change beliefs or behaviors. As shown in Figure 3, correction and prebunking, instilling cognitive feelings, and narrativization are best positioned to change beliefs, whereas the remaining interventions have been primarily developed to change behavior. However, all of them target individual factors. Thus, in the coming sections, we discuss behavioral change interventions, namely legal and administrative sanctions, increasing institutional trustworthiness, changing social norms, introducing monitors and reminders, using material incentives, increasing social support, and controlling information access (see Figures 2 and 3).

### Legal and administrative sanctions

Legal and administrative sanctions have long been advocated as behavioral change interventions. Their effects, however, are mixed. For example, mandates are effective in specific contexts, such as COVID-19 vaccination, but deterrence legal measures generally exhibit only negligible or even negative effects on behavior (Albarracín, Fayaz-Farkhad, & Granados Samayoa, 2024). Moreover, combating behaviors in areas where conspiracy beliefs are common introduces an additional challenge (Albarracín, 2020; van Prooijen & van Vugt, 2018). Conspiracy beliefs often revolve around cover-ups orchestrated by powerful hidden groups (Albarracín et al., 2021). Hence, legal interventions may inadvertently reinforce conspiratorial narratives, as regulatory actions can be interpreted by conspiracy adherents as further evidence of the alleged cover-up, thereby deepening conspiratorial thinking.

Furthermore, global regulatory efforts for misinformation have proliferated. Many countries have enacted legal and administrative sanctions for misinformation, yet these interventions are difficult to enforce (Ang et al., 2023). Limiting the production or dissemination of false information is no guarantee of behavior or belief change (Harcourt, 2021). In fact, the tangible impacts of these restrictions remain to be ascertained.

### Social norm interventions

Social norms have long been recognized as critical switches to change human behavior. Two primary types of norms have been extensively studied: injunctive norms, which refer to perceptions of what others approve or disapprove of (Fishbein & Ajzen, 2010), and descriptive norms, which reflect perceptions of what others commonly do (Cialdini et al., 1990). Interventions harnessing these norms have shown small behavioral effects in areas such as alcohol consumption, smoking, and sexual practices (Albarracín, Fayaz-Farkhad, & Granados Samayoa, 2024).

When it comes to changing behavior, normative interventions often require rectifying the perception of prevailing norms or encouraging silent majorities to speak up. For instance, college students’ alcohol use can be due to pluralistic ignorance (Prentice & Miller, 1993). They mistakenly perceive binge drinking to be more socially accepted than it is and act on this perceived norm despite personal discomfort. In such cases, normative interventions can reveal the *actual* norm and correct these misperceptions, ultimately changing behavior (Prentice & Miller, 1993; Schroeder & Prentice, 1998). For example, most Republicans support vaccination, but a vocal minority disseminates anti-vaccine views (Dixon et al., 2024). Therefore, some normative interventions encourage individuals with accurate beliefs to speak up to correct public misinformation. For example, normative messages that recommend reporting misleading material on social media, along with metrics about how many people do so, increase users’ flagging of fake news (Gimpel et al., 2021).

Normative interventions can also influence belief in misinformation. For example, providing individuals with comparative information about their own vaccine conspiracy beliefs, their perception of the public’s belief, and the actual public’s belief, along with the message that most parents vaccinate their children, reduces vaccine conspiracy beliefs and increases vaccination intentions by shaping perceived norms (Cookson et al., 2021; Ecker, Sanderson, et al., 2022). Similarly, having corrections delivered by peers or social contacts changes beliefs more than having unfamiliar or institutional sources (Bode et al., 2024; Tang et al., 2025).

Having members of one’s networks correct misinformation is a promising intervention to change beliefs. Such corrections leverage the social influence of ordinary information users, challenging misinformation directly within familiar and trusted circles. Moreover, this type of correction can have ripple effects beyond the immediate interactions within a network. When recipients observe others correcting misinformation, they too can update their beliefs through *observational correction* (Vraga & Bode, 2017). This makes peer correction a scalable strategy for enhancing public understanding, as corrections can indirectly reach audiences beyond the initial recipients.

Interventions targeting descriptive norms may offer additional advantages with respect to belief change. Beliefs about others’ behaviors form shorter inferential chains, functioning similarly to outcome beliefs. For example, the belief “Most people are getting vaccinated, so the vaccine must be good” requires only one further inference—“If the vaccine is good, I should get vaccinated”—to lead directly to a behavioral intention (Granados Samayoa & Albarracín, 2025b). Because of this short inferential path, normative interventions, particularly those emphasizing descriptive norms, may be especially effective in promoting behavior change.

### Improving institutional trustworthiness

Trust is the belief and affective feeling that another person or an institution has one’s interest at heart (Lee, 2010; Mayo, 2015; Nadelson & Jorcyk, 2014). In the context of cooperation games, trust in interaction partners increases altruistic behavior (Balliet et al., 2014). In the context of organizations, trust improves team performance and citizenship behavior (De Jong et al., 2016; Legood et al., 2021). Interventions to improve institutional trustworthiness involve persuasive communications and demonstrations that authorities are competent and benevolent. Interventions to increase the trustworthiness of healthcare authorities, however, have minimal, if any, effects on behavior, even when the intervention increases trust (Jung et al., 2023). Trust interventions also appear to have minimal effects on beliefs. For example, source trustworthiness does not affect the efficacy of prebunking interventions (Bruns et al., 2024).

In addition to being introduced as a behavioral change intervention, however, trust has been leveraged to change beliefs (Albarracín et al., 2004, 2006, 2017; Durantini et al., 2006; Hovland et al., 1949; Hovland & Weiss, 1951; Kumkale et al., 2010; Kumkale & Albarracín, 2004). This makes sense because trust in government and health agencies is negatively correlated with conspiracy beliefs and trust in health agencies (Albarracín et al., 2021; Stecula et al., 2020). However, a potential complication of intervening to make an institution appear more trustworthy is the potential misuse of that trust. For example, even though trust in science contributes to the public good, messages that use scientists as a source of misinformation produce stronger beliefs when recipients trust science (O’Brien et al., 2021). As a result, instilling critical evaluation protects audiences more than instilling trust (O’Brien et al., 2021).

### Monitoring and reminders

Monitors and reminders refer to physical or digital tools designed to track behaviors and prompt individuals to act (Albarracín, Fayaz-Farkhad, & Granados Samayoa, 2024). Manual reminders, such as tracking sheets or planners, can promote health behaviors like cancer screenings, although their impact on general preventive care is modest (Shea et al., 1996). When combined with automated or digital reminders, their effectiveness increases. For instance, both manual and computer-generated reminders significantly improve colorectal cancer screening and general preventive care (Shea et al., 1996).

Monitors and reminders have also been implemented to curb the behavior of misinformation sharing, as well as beliefs. While monitoring misinformation dissemination is promising, it comprises a technological solution more than a psychological intervention (for a detailed review of this domain, see Aïmeur et al., 2023; Saeidnia et al., 2025). However, psychologists have used accuracy prompts, which are brief interventions encouraging users to consider the accuracy of content before sharing it (Pennycook et al., 2021). This approach is based on an inattention account, which suggests that people often believe and share misinformation not due to a lack of reasoning ability or partisan bias, but because they ignore accuracy at the time (Pennycook et al., 2021). In such cases, a simple reminder to focus on accuracy is often sufficient to improve discernment and the sharing of factual information (Pennycook et al., 2021; Pennycook & Rand, 2019, 2022). Meta-analytic evidence supports this view, showing that the motivations for sharing social media information involve altruism, socialization, and entertainment. In many cases, though not all, people share misinformation with benevolent intentions. Thus, the motivational structure of misinformation sharing is not different from the motivations of information sharing more generally (Sun & Xie, 2024).

Reminders thus offer a general, low-effort intervention to address at least some beliefs. An important insight from our perspective is that specifying the targeted behavior is crucial to changing behavior (Fishbein & Ajzen, 2010; Granados Samayoa & Albarracín, 2025b). Ample evidence indicates that accuracy discernment and sharing decisions are often disconnected, even when individuals acknowledge the importance of sharing only accurate information (Arechar et al., 2023; Pennycook & Rand, 2022). Given the importance of behavioral goals, we recommend that, rather than issuing generic prompts to pay attention to accuracy, platforms provide prompts that explicitly focus on sharing behavior. This approach has been discontinued in critical social media platforms but could help reduce the inattentive sharing of misinformation.

### Material incentives

Behaviorist principles have long suggested tying desirable behavior to incentives, such as paying people to vaccinate (Baum, 1994). Many countries used financial incentives to encourage COVID-19 vaccination. A meta-analysis of the effects of these interventions showed some effects on vaccination, albeit small (Liu et al., 2024). Similarly, material incentives have been documented for energy consumption and substance use (Bolívar et al., 2021; Greene et al., 2023).

In addition, material incentives have been suggested for combatting belief in misinformation, with mixed results. On the one hand, experiments with conservative and liberal Americans showed that incentives do not correct ideological differences or misconceptions (Stein et al., 2024). In fact, increasing monetary stakes produce no belief change even when the rewards increase (Stein et al., 2024). On the other hand, monetary bonuses help when people rate the scientific validity of online misinformation (Ronzani et al., 2024). Presumably, as ideological differences have a moral basis, incentives produce resistance in the political domain. However, more objective and instrumental domains provide flexibility for incentives to exert an effect.

### Introducing social support

Social support interventions involve the provision of informational, instrumental, or financial help to a person (see Albarracín, Fayaz-Farkhad, & Granados Samayoa, 2024). They have been examined in relation to not only stress and health, but also behaviors, especially difficult behaviors that need external advice or assistance. For example, patients with cohesive families are more likely to adhere to medical treatments than those with high-conflict families (DiMatteo, 2004). Having support from family as well as exercise-class leaders and classmates increases physical activity (Carron et al., 1996). Likewise, interventions that provide social support can make weight loss programs more effective, improve medical adherence, and increase conservation behavior (Abrahamse & Steg, 2013; Bergquist et al., 2023; Lemstra et al., 2016; Shushtari et al., 2022).

Social support interventions can be used to support individuals in changing beliefs or behaviors. We already mentioned how corrections presented by peers can be effective at changing beliefs (Bode et al., 2024). Furthermore, the presence of supportive others is useful in extreme cases of malicious indoctrination. For example, people are more likely to join a cult when the cult provides unconditional acceptance and support (Rousselet et al., 2017). Cult members are also more likely to leave a cult when a supportive group outside the cult can sustain them during the transition (Rousselet et al., 2017).

### Increasing access

Access interventions can influence behavior in profound ways. For example, access to vaccination sites is a critical predictor of vaccination (Mazar et al., 2023). Likewise, the Affordable Health Care Act had tangible effects on medication use (Fayaz Farkhad et al., 2021). Specifically, United States counties in states with Medicaid expansions had greater increases in the use of pre-exposure prophylaxis to prevent HIV (Human Immunodeficiency Virus). Also, even though having health insurance, a critical resource to access health care, affects beliefs and attitudes, its direct influence on behavior remains overwhelming (Fayaz Farkhad et al., 2022).

Access interventions may also change beliefs, as in the case of access to misinformation (Jung et al., 2024). In particular, the pool of information available on social media is algorithmically curated for users. These algorithms personalize newsfeeds based on a person’s interests, potentially limiting the available information (Dubois & Blank, 2018; Grimes, 2017; Guess et al., 2018; Roberts, 2019). However, an in-depth review of this content showed that the structural influence of these algorithms is not monolithic (Lorenz-Spreen et al., 2023). On the one hand, social media users find like-minded others and form homogeneous networks (see Cota et al., 2019; Guerrero-Solé & Lopez-Gonzalez, 2017; Koiranen et al., 2019). On the other hand, social media diversifies the information people can access (Fletcher & Nielsen, 2018; Lorenz-Spreen et al., 2023; Strauß et al., 2020; Yang et al., 2020). Supporting this dichotomy, studies analyzing digital traces do find a predominance of attitude-supportive information on social media (see Bessi et al., 2016; Terren & Borge-Bravo, 2021; Williams et al., 2015), but studies of self-reported beliefs have shown that heavier social media users can be less ideologically aligned beliefs than those of lighter users (DeMora et al., 2024).

## DISCUSSION

At the outset of this review, we challenged the prevailing assumption that misinformation must be corrected to change behavior. The average belief–behavior correlation is modest and variable, and changing behavior requires a lot more than changing beliefs. Accordingly, a belief may not be a reliable driver of behavior unless specific psychological conditions are met—namely, when behavioral goals are activated, when inferential paths are short, and when beliefs remain connected to downstream decision processes and, ultimately, behavior (see Figure 1).

The framework and questions we presented (see Figures 1–3) compel a fundamental rethinking of intervention strategies in the misinformation domain. Rather than approaching belief correction as a default solution, we propose a more targeted, function-driven logic: one that begins by identifying the intended outcome—belief or behavior change—and selects intervention strategies accordingly. For example, journalists may still prioritize belief correction even if behavioral consequences are minimal. But when the primary objective is to change behavior, interventions that use correcting or prebunking should focus on outcome beliefs, emphasize behavioral relevance, or include more direct calls to action. Interventions may also be selected because they are designed to change behavior (see Figure 3).

When belief-change interventions are used to change behavior, they should account for the temporal dynamics of belief-to-behavior inferences. Beliefs formed in the absence of behavioral goals may appear inert but could be reactivated at critical decision points, exerting a delayed influence on behavior. In such cases, preventive approaches like prebunking, which prepare people to resist misinformation before it is encoded, may be especially effective. Prebunking strategies that train behavioral skills may be particularly effective by fostering information scrutiny. With reduced scrutiny, the misinformation may not be integrated into a belief-to-behavior inference to begin.

Importantly, our review introduced several promising alternatives to correction and prebunking, particularly when the goal is to change behavior. Bypassing, for instance, redirects attention to alternative, behaviorally consequential beliefs without directly challenging existing misconceptions. Self-affirmation reduces defensiveness in identity-relevant contexts, while motivational interviewing may enable individuals to explore their own reasons for change. These approaches share a key feature: they shift the locus of change from belief accuracy to behavior change.

Beyond individual-level strategies, social-structural interventions expand the range of possibilities for behavior, and possibly belief change as well. Social norms, reminders, social support, and access mechanisms can modify behavioral contexts in important ways. These contextual influences can shape behavior directly. In addition, they may change beliefs, as is the case when a supportive social network allows cult members to leave a cult.

In sum, even though this paper is primarily conceptual, our framework has several practical implications. Figure 3 depicts these implications, showing strategies best suited for belief change in red and those appropriate for behavior change in blue. To change a belief, correction and prebunking, cognitive feelings, and perhaps narrativization all offer possibilities. Changing a belief might also affect behavior when people form it or retrieve it along with a behavioral goal. This is most likely to occur when the belief concerns a behavioral outcome, is introduced along with a behavioral recommendation, or is activated in a behaviorally relevant context (Albarracín et al., 2018; see Figure 1). Across the board, however, interventions targeting beliefs should have negligible effects on behavior (Albarracín, Fayaz-Farkhad, & Granados Samayoa, 2024).

To change behavior, bypassing, self-affirmation, social normative interventions, monitors and reminders, and material incentives, should each receive close attention. Notably, behavioral-skills training, social-norm interventions, monitors and reminders, and material incentives have small effects as well. However, the most effective interventions according to Albarracín, Fayaz-Farkhad, and Granados Samayoa’s (2024) estimates are instilling habits and introducing social support, both of which have medium behavioral effects, as well as increasing access to opportunities to perform a behavior, which has large effects. Meanwhile, legal and administrative sanctions and interventions to increase institutional trustworthiness have negligible effects on average.

In closing, it is critical to choose intervention strategies based on the cognitive architecture of belief–behavior correspondence and the desire to change beliefs or behavior. Changing beliefs can serve to change behavior—but only when beliefs are behaviorally engaged, inferentially accessible, and contextually relevant. Recognizing these boundary conditions enables more precise, theory-informed interventions with measurable effects. By focusing not only on what people believe but also on the broader forces driving behavior, future interventions may be better calibrated to the realities of human cognition. The fight against misinformation is not just about educational efforts. Interventions must go beyond identifying what is false and recognize when, how, and why a belief matters for behavior, and when behavior must be addressed in a direct way.

## Data Availability

Data sharing not applicable to this article as no datasets were generated or analysed during the current study.
